# Partner smoking and maternal cotinine during pregnancy: Implications for negative control methods^☆^

**DOI:** 10.1016/j.drugalcdep.2014.03.012

**Published:** 2014-06-01

**Authors:** Amy E. Taylor, George Davey Smith, Cristina B. Bares, Alexis C. Edwards, Marcus R. Munafò

**Affiliations:** a MRC Integrative Epidemiology Unit (IEU) at the University of Bristol, UK Centre for Tobacco and Alcohol Studies, School of Experimental Psychology, University of Bristol, 12a Priory Road, Bristol BS8 1TU, UK; b MRC Integrative Epidemiology Unit (IEU) at the University of Bristol, School of Social and Community Medicine, University of Bristol, Oakfield House, Oakfield Grove, Bristol BS8 2BN, UK; c School of Social Work, Virginia Commonwealth University, Richmond, VA 23284-2027, USA; d Virginia Institute for Psychiatric and Behavioral Genetics, Department of Psychiatry, Virginia Commonwealth University, Richmond, VA 23298-0126, USA

**Keywords:** ALSPAC, Cigarette smoking, Pregnancy, Intrauterine exposure, Negative control

## Abstract

**Background:**

Comparison of the associations of maternal and mother's partner smoking with offspring outcomes is, in theory, a useful method for assessing whether there may be an intrauterine effect of tobacco exposure on these outcomes. However, this approach assumes that the effects of passive smoking from exposure to partner smoking during pregnancy are minimal. We evaluated this assumption using a biochemical measure of tobacco exposure in pregnant women.

**Methods:**

Cotinine levels taken during the first trimester of pregnancy were measured in a sample of 3928 women from the Avon Longitudinal Study of Parents and Children. Median cotinine values were compared across categories of smoking heaviness (cigarettes per day) of the women during the first trimester and in non-smoking women by the smoking heaviness of their partner.

**Results:**

Cotinine levels were substantially higher in women who smoked compared to non-smokers (range of medians across smoking heaviness categories: 900–5362 ng/ml versus 20 ng/ml, interquartile range (IQR) (0–63) for non-smokers). In contrast, cotinine levels in non-smoking women were only very weakly related to partner smoking status (range of medians in women with smoking partners: 34–69 ng/ml versus 12 ng/ml, IQR (0–48) in women with non-smoking partners).

**Conclusions:**

Levels of tobacco exposure from partner smoking, as assessed by cotinine, were low in non-smoking pregnant women. This suggests that using mother's partner's smoking as a negative control for investigating intrauterine effects is valid.

## 1 Introduction

Maternal smoking during pregnancy has been associated with a range of adverse outcomes in offspring including decreased offspring birth weight (Tyrrell et al., 2012), lower language ability (Key et al., 2007), developmental delays, a reduction in intellectual ability (Butler and Goldstei, 1973), lower levels of executive function (Piper and Corbett, 2012), and higher rates of attention and behavioural problems (Agrawal et al., 2010; Roza et al., 2009; Stene-Larsen et al., 2009; Hutchinson et al., 2010; Nomura et al., 2010; Wakschlag et al., 2006). In addition, offspring of mothers who smoked during pregnancy have been shown to have greater risk of substance use and dependence in adolescence and adulthood (Nomura et al., 2011; Monshouwer et al., 2011; Lawlor et al., 2005; Al Mamun et al., 2006; Buka et al., 2003; Kandel et al., 1994; Munafò et al., 2006; Roberts et al., 2005).

However, the observed relationship between smoking during pregnancy and offspring outcomes may not be causal but instead due to confounding, and isolating the specific causal effects of intrauterine exposure (as opposed to the effects of parental smoking behaviour in childhood and adolescence) can be difficult. One solution is to compare the effects of smoking during pregnancy to a negative control, where no association would be expected (Davey Smith, 2008, 2012). In the case of maternal smoking during pregnancy, this can be done by comparing associations of maternal and the mother's partner's smoking during pregnancy with offspring outcomes, using mother's partner's smoking as a negative control. If there were an intrauterine effect of tobacco exposure, we would expect associations between maternal smoking and offspring outcomes to be stronger than associations between partner smoking and offspring outcomes (Davey Smith, 2008). If effects are of similar magnitude, this suggests that associations between maternal smoking during pregnancy and offspring outcomes are due to confounding, either by shared environmental or genetic factors, which may themselves be causal factors (Davey Smith, 2008), or due to a causal influence operating outside of pregnancy, such as the offspring modelling smoking behaviour in childhood and adolescence (Gilman et al., 2009). This technique has been applied previously to investigate the effects of smoking during pregnancy on offspring birth weight, blood pressure, trajectories of height and adiposity, offspring smoking initiation and attention deficit hyperactivity disorder (Brion et al., 2007; Howe et al., 2012; Taylor et al., 2014; Langley et al., 2012). Importantly, the validity of the negative control method may depend on the nature of the offspring behaviour measured, and the time between intrauterine exposure and measurement of the offspring behaviour.

A key assumption of the negative control method is that the biological effects of partner smoking on intrauterine exposure are negligible compared to those of active maternal smoking. However, it is plausible that this is not the case; several studies have demonstrated that environmental exposure to tobacco smoke is detectable in pregnant women through assessment of cotinine, a biochemical measure of tobacco exposure (Kvalvik et al., 2012; Jordanov, 1990; Rebagliato et al., 1995). Furthermore, in a recent study, partner smoking was found to be the source of tobacco exposure most strongly associated with cotinine levels in non-smoking pregnant women (Aurrekoetxea et al., 2014). Given the potential promise of negative control methods to assess the causal effects of intrauterine tobacco exposure, we aimed to compare levels of cotinine in pregnant smokers and non-smokers and to explore the association of maternal cotinine with partner smoking behaviour in the Avon Longitudinal Study of Parents and Children (ALSPAC).

## 2 Methods

### 2.1 Study population

ALSPAC is a prospective cohort study which recruited 14,541 pregnant women living in Avon, United Kingdom with expected dates of delivery between 1st April, 1991 and 31st December, 1992. These pregnancies resulted in 14,062 live births and 13,988 children who were alive at one year of age. Full details of the recruitment process have been published previously (Boyd et al., 2013; Fraser et al., 2013). Since enrolment, detailed information on the children, mothers and their partners has been collected via postal questionnaires and clinic visits. Please note that the study website contains details of all the data that are available through a fully searchable data dictionary (http://www.bristol.ac.uk/alspac/researchers/data-access/data-dictionary/). Ethics approval for the study was obtained from the ALSPAC Ethics and Law Committee and the Local Research Ethics Committee.

### 2.2 Measures

Mothers provided information about their smoking status during the first trimester of pregnancy in a questionnaire administered at 18 weeks gestation. Mothers were asked if they smoked regularly (cigarettes, pipe, cigar or other) and the number smoked per day. Mothers’ partners also provided information on their smoking status and heaviness at the start of pregnancy in a questionnaire administered at 18 weeks gestation. In addition, mothers were asked about their partner's current smoking at 18 weeks gestation. The correlation between partner self-report of smoking heaviness and maternal report of partner smoking heaviness was high (*r* = 0.89, *r* = 0.75 if restricted to smokers). A list of the smoking questions asked is provided in the supplementary material.^1^ Information on demographic characteristics of participants was obtained from questionnaires administered during pregnancy.

Cotinine levels (ng/ml) were assessed from a single urine sample taken during the first trimester of pregnancy. For most mothers the samples were collected as part of routine clinical care but some samples were obtained specifically for ALSPAC. Urine samples were stored at −20 °C and allowed to thaw at room temperature before use. Cotinine levels (expressed as nanograms per millilitre of serum) were measured in duplicate by the Cozart Cotinine Enzyme Immunoassay (Concateno UK, Abingdon) serum kit (M155B1). Absorbance was measured spectrophotometrically at a wavelength of 450 nm. Samples were diluted using cotinine-free serum (foetal calf serum) if required.

### 2.3 Statistical analysis

Analyses were restricted to non-smokers and cigarette smokers who had measured cotinine data. Mothers and partners only reporting pipe, cigar or other types of smoking were excluded. Cotinine data were strongly right skewed. Medians and interquartile ranges (IQR) of cotinine were calculated for all mothers stratified by their self-reported smoking status (non-smokers, 1–4, 5–9, 10–14, 15–19, 20–24, 25–29 and 30+ cigarettes per day). Within mothers who reported being a non-smoker during the first trimester of pregnancy, medians and interquartile ranges of cotinine were calculated stratified by: (1) partner self-report of smoking status at the start of pregnancy, and (2) maternal report of partner smoking at 18 weeks gestation. All analyses were conducted in Stata (version 11).

## 3 Results

### 3.1 Characteristics of participants

The final sample for analysis consisted of 3928 non-smoking and cigarette smoking women during the first trimester of pregnancy (see Fig. S1 in supplementary material for a flowchart of the analysis sample^2^). Mothers included in the analyses reported in >98% cases that their current partner was the father of their unborn child. According to self-reports of marital status at 12 weeks gestation, 80% of mothers and 84% of partners were married. Although there was some statistical evidence of differences between ALSPAC participants with and without maternal cotinine data in age, educational attainment and maternal smoking, the absolute magnitude of these differences was small. The characteristics of participants with and without cotinine data available are presented in Table 1.

### 3.2 Cotinine levels in smoking and non-smoking mothers

Cotinine levels were considerably higher in smoking mothers across all levels of heaviness of smoking (medians 900–5362 ng/ml) compared to non-smoking mothers (median 20 ng/ml, IQR 0 to 63). Cotinine levels increased in a roughly linear manner with self-reported heaviness of smoking. Among non-smoking mothers, median cotinine levels in mothers with smoking partners ranged from 34 to 69 ng/ml (where partner self-report of smoking was used) and from 33 to 71 ng/m (where mother report of partner smoking was used), irrespective of heaviness of partner smoking. These were only slightly higher than levels in mothers with non-smoking partners (median 12 ng/ml, IQR 0–48 where partner self-report of smoking used; median 13 ng/ml, IQR 0–50 where mother report of partner smoking used). These results are reported in Table 2.

The results of linear regression analyses (presented in supplementary material) indicated a dose-response relationship between both maternal and partner smoking heaviness and maternal cotinine levels. However, the strength of the association between active smoking and maternal cotinine was an order of magnitude higher than for partner smoking and maternal cotinine in non-smoking mothers.

## 4 Discussion

Our results confirm that the levels of cotinine present in non-smoking mothers with smoking partners are two orders of magnitude lower than the levels present in smoking mothers. The clear difference in tobacco exposure between active smoking mothers and non-smoking mothers potentially exposed to passive smoking supports the assumption of negative control that the effects of passive smoking from exposure to partner smoking during pregnancy are likely to be minimal in comparison to active smoking.

There are some limitations to the data presented here. Cotinine data were only available for the first trimester of pregnancy so these results may not be generalizable to the whole of pregnancy. In addition, we do not know how exposed the mothers were to passive smoking during the first trimester if their partner smoked, or whether they might have been exposed to passive smoking from other household members at this time. Mothers were asked about passive smoke exposure in a questionnaire administered at 18 weeks gestation, and restricting analyses to mothers reporting passive smoke exposure of ≥1 h per day did not alter our results (data not shown). However, these results may not be generalizable to populations with different patterns of passive smoking exposure. Finally, these data were not specifically collected for the purposes of answering this question and the analyses should be interpreted in this context.

We cannot exclude the possibility of a biological effect of partner smoking on offspring outcomes. Environmental tobacco smoke (ETS) exposure (sometimes described as secondhand smoke exposure or “passive smoking”) is acknowledged to have detrimental health consequences, such as elevated risk for cardiovascular disease, lung cancer, and respiratory disease (Royal-College-of-Physicians, 2010), and therefore has biological effects. However, in our opinion it is implausible that these very low levels of exposure would have intrauterine effects comparable to those resulting from the exposure levels associated with active smoking by the mother. In other words, we can be confident that if similar associations are observed between maternal and partner smoking and offspring outcomes, this is most parsimoniously explained by residual confounding rather than a direct causal effect of partner smoking on foetal development.

Determining the causal nature of observed associations between exposures such as maternal smoking during pregnancy and offspring outcomes, is critical if appropriate targeted interventions are to be developed. A number of approaches to determining causality exist, each with their own strengths and limitations. For example, Mendelian randomization leverages genetic associations with environmental exposures while protecting against reverse causality and (under certain assumptions) confounding (Ebrahim and Davey Smith, 2008). The identification of variants associated with smoking cessation, including during pregnancy, provides an opportunity to implement this technique (Freathy et al., 2009; Munafò et al., 2011), and this approach has been successfully applied to establish the effects of continuing to smoke during pregnancy on offspring birth weight (Tyrrell et al., 2012). However, the magnitude of the effects of common genetic variants on complex behaviour outcomes such as smoking during pregnancy means that single genetic variants are weak instruments. This means that large samples are required, and these may be difficult to obtain in practice. Therefore, the use of negative control may be a useful alternative.

We have shown, in a large population sample, that the association of mother's partner smoking with cotinine levels in non-smoking pregnant mothers is small in comparison to the effects of active smoking. This confirms the validity of using partner smoking during pregnancy as a negative control method for investigating the effects of maternal smoking during pregnancy. Where these data are available, this provides a potentially powerful method for determining whether observed associations are likely to be causal.

## Role of funding source

AET and MRM are members of the United Kingdom Centre for Tobacco and Alcohol Studies, a UKCRC Public Health Research: Centre of Excellence. Funding from British Heart Foundation, Cancer Research UK, Economic and Social Research Council, Medical Research Council, and the National Institute for Health Research, under the auspices of the UK Clinical Research Collaboration, is gratefully acknowledged. This work was supported by the Wellcome Trust (grant number 086684) the NIH (grant number AA021399), NIDA (grant number R25DA030310), and the Medical Research Council (grant numbers MR/J01351X/1, G0800612, G0802736, MC_UU_12013/1-9). The funders had no further role in study design; in the collection, analysis and interpretation of data; in the writing of the report; or in the decision to submit the paper for publication.

## Contributors

MRM and GDS came up with the concept of the paper. AET performed the statistical analysis. MRM and AET wrote the first draft of the manuscript. All authors contributed to and have approved the final manuscript.

## Conflict of interest

The authors have no conflicts of interest to declare.

## Figures and Tables

**Table 1:** Comparison of samples with and without maternal cotinine data in ALSPAC.

	Maternal cotinine data available				*P*-value^b^
	Yes		No		
	Mean	SD	Mean	SD	
Maternal age (years)^a^	28.2	(4.8)	27.6	(5.0)	<0.001
	*N*	%	*N*	%	
Maternal education^c^					<0.001
CSE	670	(17.6)	1783	(21.4)	
Vocational	363	(9.5)	835	(10.0)	
O level	1360	(35.7)	2833	(34.1)	
A level	902	(23.7)	1812	(21.8)	
Degree	510	(13.4)	1053	(12.7)	

Socioeconomic position^d^					0.19
I	369	(10.8)	807	(11.1)	
II	1193	(34.7)	2437	(33.7)	
III (non-manual)	395	(11.5)	756	(10.4)	
III (manual)	1067	(31.1)	2298	(31.7)	
IV	326	(9.5)	724	(10.0)	
V	84	(2.5)	221	(3.1)	

Maternal smoking^e^					<0.001
None	3099	(78.9)	6588	(74.0)	
1–4	195	(5.0)	482	(5.4)	
5–9	199	(5.1)	517	(5.8)	
10–14	205	(5.2)	570	(6.4)	
15–19	113	(2.9)	379	(4.3)	
20–24	87	(2.2)	265	(3.0)	
25+	30	(0.8)	104	(1.2)	

Partner smoking^e^^,^^f^					0.12
None	1867	(68.8)	3839	(65.7)	
1–4	82	(3.0)	178	(3.1)	
5–9	92	(3.4)	220	(3.8)	
10–14	163	(6.0)	353	(6.0)	
15–19	196	(7.2)	452	(7.7)	
20–24	207	(7.6)	511	(8.8)	
25–29	52	(1.9)	149	(2.6)	
30+	53	(2.0)	139	(2.4)	

a *N* for maternal age analyses 3946 with cotinine data and 8982 without cotinine data.

b *P*-values from chi square test for categorical variables and *t*-test for continuous variables.

c CSE, vocational and O-level are qualifications taken at 16 years and A-levels are examinations taken at 18 years. Qualifications are listed in ascending order of educational attainment.

d Classification of socioeconomic position based on partner's occupation according to United Kingdom Office of Population Census and Surveys (OPCS), with I representing the highest socioeconomic position. Details of these classifications in ALSPAC have been reported previously (Farrow et al., 1998).

e Cigarette smoking only. Excludes cigar, pipe and other smokers.

f Partner report of smoking heaviness.

**Table 2:** Cotinine levels in all mothers by smoking status, and non-smoking mothers by partner smoking status.

All mothers^a^ *N* = 3928					Non-smoking mothers^b^ *N* = 2216					Non-smoking mothers^c^ *N* = 2800				
Mother Smokes (cig/day)	*N*	%	Maternal Cotinine (ng/ml)		Partner Smokes (cig/day)	*N*	%	Maternal Cotinine (ng/ml)		Partner Smokes (cig/day)	*N*	%	Maternal Cotinine (ng/ml)	
			Median	IQR				Median	IQR				Median	IQR
None	3099	78.9	20	0–63	None	1704	77.0	12	0–48	None	2204	78.7	13	0–50
1–4	195	5.0	900	76–2497	1–4	59	2.7	34	0–72	1–4	71	2.5	37	0–78
5–9	199	5.1	2214	891–4375	5–9	64	2.9	42.5	4–157.5	5–9	81	2.9	43	8–116
10–14	205	5.2	3030	1180–4729	10–14	104	4.7	39.5	0–107	10–14	127	4.5	38	0–90
15–19	113	2.9	3062	1584–5907	15–19	115	5.2	61	0–179	15–19	135	4.8	43	0–130
20–24	87	2.2	3657	1722–6132	20–24	115	5.2	56	21–115	20–24	113	4.0	71	32–203
25+^d^	30	0.8	5362	1697–7309	25–29	28	1.3	69	3–250.5	25–29	41	1.5	33	0–195
					30+	27	1.2	52	0–146	30+	28	1.0	40.5	6–129.5

a Cigar, pipe or other smoking excluded (*N* = 14).

b Partner self-report of smoking heaviness at the start of pregnancy. Cigar, pipe or other smoking excluded (*N* = 123).

c Mother's report of partner's smoking heaviness at 18 weeks of pregnancy. Cigar, pipe or other smoking excluded (*N* = 184).

d 25–29 and 30+ categories combined due to small numbers in the 30+ category.

## Footnotes

1 Supplementary material can be found by accessing the online version of this paper. Please see Appendix A for more information.

2 Supplementary material can be found by accessing the online version of this paper. Please see Appendix A for more information.
