# Supplementary material for: Partner smoking and maternal cotinine during pregnancy: Implications for negative control methods
Source: Drug Alcohol Depend. 2014 Jun 1;139(100):159–63. doi: 10.1016/j.drugalcdep.2014.03.012 (PMC4026952; doi:10.1016/j.drugalcdep.2014.03.012)
Supplement: Supplementary file 1 [file mmc1.docx]

**Supplementary Material for the Article**

Partner smoking and maternal cotinine during pregnancy: implications for negative control methods

Amy E. Taylor ^1^, George Davey Smith ^2^, Cristina B. Bares ^3^, Alexis C. Edwards ^4^, Marcus R. Munafò ^1^

1. MRC Integrative Epidemiology Unit (IEU) at the University of Bristol, UK Centre for Tobacco and Alcohol Research Studies, School of Experimental Psychology, University of Bristol, 12a Priory Road, Bristol, BS8 1TU

2. MRC Integrative Epidemiology Unit (IEU) at the University of Bristol, School of Social and Community Medicine, University of Bristol, Oakfield House, Oakfield Grove, Bristol, BS8 2BN

3. School of Social Work, Virginia Commonwealth University, Richmond, Virginia 23284-2027, USA.

4. Virginia Institute for Psychiatric and Behavioral Genetics, Department of Psychiatry, Virginia Commonwealth University, Richmond, Virginia 23298-0126, USA

**Corresponding Author:**

Amy Taylor

MRC Integrative Epidemiology Unit, UK Centre for Tobacco and Alcohol Studies, and School of Experimental Psychology

University of Bristol

Bristol BS8 1TU

UK

T: +44.117.9288547

F: +44.117.9288588

E: [amy.taylor@bristol.ac.uk](mailto:amy.taylor@bristol.ac.uk)

**This material supplements, but does not replace, the peer-reviewed article in**

***Drug and Alcohol Dependence.***

**Smoking questions in ALSPAC**

**Maternal smoking in first trimester**

**At 18 weeks gestation, mothers were asked:**

*Did you smoke regularly in the first three months of pregnancy?*

*No*

*Yes, cigarettes*

*Yes, cigars*

*Yes, pipe*

*Yes, other (please describe)*

*How many times per day did you smoke in the first 3 months of your pregnancy?*

*0*

*1-4*

*5-9*

*10-14*

*15-19*

*20-24*

*25-29*

*30+*

**Partner smoking in first trimester**

**At 18 weeks gestation, mothers were asked:**

*Does your partner smoke?*

*No*

*Yes, cigarettes*

*Yes, cigars*

*Yes, pipe*

*Yes, other (please describe)*

*If yes, about how many times per day does your partner smoke at the moment?*

*0*

*1-4*

*5-9*

*10-14*

*15-19*

*20-24*

*25-29*

*30+*

**At 18 weeks gestation, partners were asked:**

*How many times per day did you smoke at the start of your partner's pregnancy?*

*0*

*1-4*

*5-9*

*10-14*

*15-19*

*20-24*

*25-29*

*30+*

Figure S1. Flowchart of study population

14,541 pregnant women recruited

3,928 with complete data for analysis

14,202 women excluding multiple pregnancies

4,137 with measured cotinine

**Additional statistical analysis**

**Methods**

A Kruskal Wallis test was performed to test for statistical evidence for a difference between cotinine levels in smoking and non-smoking mothers. Within smoking mothers, linear regression was used to assess the association between maternal and partner smoking heaviness (treated as linear variables) and maternal cotinine levels in smoking mothers, first separately and then in a mutually adjusted model. Likelihood ratio tests were used to assess evidence for non-linear relationships between smoking heaviness variables and maternal cotinine levels. Linear regression was also performed in the sample of non-smoking mothers to assess associations between paternal smoking heaviness and cotinine levels. For all analyses robust standard errors were used to account for potential non normality of residuals.

**Results**

**Table S1. Comparison of cotinine levels in smoking and non-smoking mothers:**

|  | **N** | **Median** | **(IQR)** |
| --- | --- | --- | --- |
| **Non-smoking mothers** | 3,099 | 20 | (0,63) |
| **Smoking mothers** | 829 | 2,460 | (861, 4,556) |

Kruskal Wallis test of difference: chi-squared = 1470.046, probability = 0.0001.

**Table S2. Linear regression analysis of association between mother and partner smoking and maternal cotinine levels in *smoking mothers (*N=707)**

|  | **Effect on maternal cotinine (ng/ml) per cigarette per day** | **95% CI** | **P-value** |
| --- | --- | --- | --- |
| **Maternal heaviness** | 139 | (97, 181) | <0.001 |
| **Partner heaviness** | 47 | (19, 74) | 0.001 |
| **Maternal heaviness (adjusted for partner)** | 131 | (87, 175) | <0.001 |
| **Partner heaviness (adjusted for maternal)** | 30 | (1,58) | 0.04 |

Smoking heaviness measured as a categorical variable (1,5,10,15,20,25 for mother and 0,1,5,10,15,20,25,30 for partner) but treated as a continuous variable as there was no clear statistical evidence for non-linearity. Partner heaviness was assessed from mother report.

**Table S3. Linear regression analysis of association between partner smoking and maternal cotinine levels in *non-smoking mothers (*N=2,800)**

|  | **Effect on cotinine (ng/ml) per cigarette per day** | **95% CI** | **P-value** |
| --- | --- | --- | --- |
| **Partner heaviness** | 8.3 | (3.9, 12.7) | <0.001 |

Smoking heaviness measured as a categorical variable (0,1,5,10,15,20,25,30) but treated as a continuous variable as there was no clear statistical evidence for non-linearity. Partner heaviness was assessed from mother report.
